# A man with small vessel vasculitis presenting with brachial diplegia, multiple cranial mononeuropathies and severe orthostatic hypotension in diabetes mellitus: a case report

**DOI:** 10.1186/1752-1947-7-229

**Published:** 2013-10-01

**Authors:** Sahar F Zafar, Jerry Clay Goodman, Eroboghene E Ubogu

**Affiliations:** 1 Department of Neurology, Baylor College of Medicine, One Baylor Plaza, MS NB 302, Houston, TX 77030, USA; 2 Department of Pathology and Immunology, Baylor College of Medicine - BCM 315, One Baylor Plaza, Houston, TX 77030, USA

**Keywords:** Brachial diplegia, Diabetes mellitus, Electrophysiology, Mononeuropathy multiplex, Nerve biopsy, Orthostatic hypotension, Peripheral neuropathy, Vasculitis

## Abstract

**Introduction:**

We report a rare case of fulminant vasculitic mononeuropathy resulting in brachial diplegia, with suspected brainstem and autonomic nervous system involvement in a patient with diabetes mellitus.

**Case presentation:**

A 58-year-old Hispanic Caucasian man with diabetes mellitus presented with a 1-year history of progressive bilateral upper extremity weakness, orthostatic intolerance and progressive memory decline. Diagnostic evaluation including laboratory tests for progressive encephalopathies, systemic inflammatory and non-inflammatory neuropathies, cerebrospinal fluid analyses, electrodiagnostic studies, and nerve biopsy were performed. Clinical examination revealed moderate cognitive deficits on the Montreal Cognitive Assessment scale, bilateral facial weakness and weakness of bilateral shoulder girdle and intrinsic hand muscles. Cerebrospinal fluid analyses revealed elevated protein and an elevated immunoglobulin G synthesis rate, suggesting an immune-mediated process. Further laboratory work up was non-diagnostic. Electrodiagnostic studies demonstrated chronic asymmetric axonal mononeuropathies with ongoing denervation. A superficial radial nerve biopsy showed a chronic vasculitic neuropathy. Glucocorticosteroid treatment, symptomatic pharmacologic and supportive non-pharmacologic therapies resulted in improved clinical outcomes despite challenges with glycemic control.

**Conclusions:**

This case report emphasizes the importance of a thorough evaluation of atypical or uncommon neuromuscular presentations in diabetic patients without etiological presumptions. This is necessary in order to promptly establish a diagnosis, initiate appropriate therapies and prevent irreversible nerve injury.

## Introduction

Diabetes mellitus is a known cause of peripheral nervous system (PNS) disease. Common PNS manifestations include length-dependent distal sensory and sensorimotor polyneuropathies (commonly known as 'diabetic neuropathy’). Other neurologic manifestations include mononeuropathies such as carpal tunnel syndrome, autonomic neuropathies and slow cognitive decline [1,2]. Multiple mononeuropathies (or mononeuropathy multiplex) is an uncommon form of neuropathy in diabetics; usually attributed to an immune-mediated process responsive to immunosuppressants [3]. A more common cause for mononeuropathy multiplex is peripheral nerve vasculitis [4-6]. This disorder may manifest clinically as an asymmetric sensorimotor polyneuropathy that can be quite fulminant depending on the aggressiveness of the inflammatory process. Less frequent presentations include sequential cranial neuropathies, autonomic neuropathy, pure sensory neuropathies and rapid cognitive decline [7-9].

We report the case of a patient who presented with severe progressive, asymmetric bilateral upper extremity weakness (also known as brachial diplegia), associated with rapid onset cognitive decline, cranial mononeuropathies and orthostatic hypotension, in the setting of suboptimal diabetes mellitus control. Upper extremity involvement while sparing the lower extremities, facial nerve involvement and the rapid cognitive decline are atypical features of diabetic neuropathy; providing the rationale to investigate for an alternative etiology. Electrophysiological testing was consistent with chronic multiple axonal mononeuropathies with ongoing denervation, consistent with multiple mononeuropathies. Peripheral nerve histopathological evaluation demonstrated small vessel vasculitis.

This case report suggests the importance of evaluating for peripheral nerve vasculitis in diabetic patients with non-length-dependent or multiple mononeuropathies, particularly if restricted to the upper extremities, rather than assume a metabolic microangiopathy. Vasculitis is a pathological finding that significantly alters the clinical management of PNS disease. Failure to diagnose and treat peripheral nerve vasculitis may result in irreversible axonal injury with associated significant morbidity. In a patient with a combination of vasculitis and diabetes, special care and close medical follow up is required when administering immunosuppressants, particularly corticosteroids. Weighing risks associated with systemic immunosuppression with the potential benefits associated with accelerated peripheral nerve recovery should be performed to maximize the chances for positive patient outcomes.

## Case presentation

A 58-year-old Hispanic Caucasian man with diabetes mellitus presented to the Emergency Center with a 1-year history of progressive bilateral upper extremity weakness and episodes of orthostatic lightheadedness. He initially noticed weakness in his right-hand grip that gradually progressed over the next 6 to 8 months to involve the left hand and eventually both arms, to the extent that he was unable to hold objects or elevate his arms. He also complained of a tingling and burning sensation in both hands. His family had noticed mild bilateral facial weakness, described as reduced facial expression, without dysphagia or dysarthria. He did not complain of any lower extremity weakness or sensory symptoms. There was no bowel or bladder dysfunction, and he denied any erectile dysfunction. Prior to evaluation, he had been experiencing orthostatic intolerance that worsened to the point that he became non-ambulatory. His family had also noticed cognitive decline over the last year, with frequent forgetfulness and slow thought processing. On systems review, he reported mild xerostomia and xerophthalmia without dysphagia.

A general physical examination, including cardiovascular, respiratory and abdominal systems, was normal. On initial neurological examination, he was awake, alert and oriented to person, place, time and situation. He had a Montreal Cognitive Assessment (MoCA) score of 16 out of 30, with deficits primarily in the visuospatial, executive and delayed recall domains. On cranial nerve examination, he had preserved pupillary responses, visual fields were full on confrontational testing, and he had normal fundoscopy bilaterally. His extraocular movements were preserved. He had bilateral facial weakness (facial diplegia) and decreased subjective sensation to light touch and pinprick in the left trigeminal nerve distribution. He did not have any hearing impairment and his uvula and palate elevated symmetrically. He did not have any weakness in his sternocleidomastoid, trapezius or tongue muscles. On motor testing, there was decreased tone in his upper extremities, with bilateral shoulder girdle and intrinsic hand muscle atrophy. On confrontational strength testing (based on the six-point Medical Research Council scale), he had normal neck flexion and extension strength. He had near symmetric proximal and distal weakness in the upper extremities, with strength of two to three out of five in all muscle groups tested, slightly worse on his right (Table 1). His strength was normal in his lower extremities.

**Table 1:** Serial confrontational motor strength testing, Montreal cognitive assessment testing, and orthostatic vitals

**Muscle group**	**Muscle strength grade – data on initial examination**	**Muscle strength grade – data at 3-month follow up**	**Muscle strength grade – data at 1-year follow up**
Shoulder flexors	2/5 (L), 3/5 (R)	2/5 (L), 3/5 (R)	3/5 (L), 4/5 (R)
Shoulder extensors	2/5 (L), 2/5 (R)	2/5 (L), 2/5 (R	4/5 (L), 5/5 (R)
Shoulder abductors	2/5 (L), 2/5 (R)	2–/5 (L), 2/5 (R)	2/5 (L), 5/5 (R)
Shoulder adductors	3/5 (L), 3/5 (R)	3/5 (L), 3/5 (R)	4/5 (L), 5/5 (R)
Elbow flexors	3–/5 (L), 3/5 (R)	3–/5 (L), 3/5 (R)	4/5 (L), 5/5 (R)
Elbow extensors	3/5 (L), 3/5 (R)	4–/5 (L), 4+/5 (R)	3+/5 (L), 4+/5 (R)
Wrist flexors	2/5 (L), 2/5 (R)	3/5 (L), 4/5 (R)	3/5 (L), 4/5 (R)
Wrist extensors	2/5 (L), 2/5 (R)	3–/5 (L), 4/5 (R)	3/5 (L), 4/5 (R)
Finger flexors	2/5 (L), 3/5 (R)	2+/5 (L), 4–/5(R)	3+/5 (L), 4/5 (R)
Finger extensors	3/5 (L), 3/5 (R)	2+/5 (L), 4/5(R)	3/5 (L), 3+/5 (R)
Finger abductors	2/5 (L), 3/5 (R)	2/5 (L), 3/5 (R)	2/5 (L), 3+/5 (R)
Finger adductors	3/5 (L), 3/5 (R)	3/5 (L), 3/5 (R)	3/5 (L), 3/5 (R)
**MoCA score**	16/30	16/30	16/30
**Orthostatic vitals**			
Supine	BP 142/90, pulse 68	BP 143/96, pulse 71	Not recorded
Sitting	BP 97/64, pulse 77	BP 128/84, pulse 72	BP 132/88, pulse 73
Standing	BP 65/40, pulse 81	BP 98/62, pulse 74	BP 102/70; pulse 83

Muscle strength data is based on the six-point Medical Research Council scale (0, no movement; 1, some movement with an inability to move the joint; 2, movement of the joint but inability to overcome gravity; 3, ability to overcome gravity but not passive resistance; 4, ability to overcome partial resistance but not full resistance; 5, normal strength).

Key: +, indicates slightly more; – indicates slightly less; BP, blood pressure (measured in mmHg); L, left; MoCA, Montreal Cognitive Assessment; R, right. Pulse measured in beats per minute.

On multimodal sensory examination, he had subjectively decreased sensation to light touch and pinprick in his left radial nerve, left median nerve and right axillary nerve distributions. A sensory examination of his lower extremities was normal. His triceps and patellar reflexes were diminished bilaterally, with preservation of his other myotactic stretch reflexes. His plantar responses were flexor bilaterally, and he did not demonstrate any frontal cortical release signs. Automated blood pressure and heart rate measurements performed at the bedside with postural change demonstrated severe orthostatic hypotension with sympathetic α- and β-adrenergic compromise as follows: supine blood pressure, 142/90mmHg (mean arterial pressure, MAP, 107mmHg) with heart rate 68 beats/minute; sitting blood pressure, 97/64mmHg (MAP 75mmHg) and heart rate 77 beats/minute; standing blood pressure, 65/40mmHg (MAP 48mmHg) and heart rate: 81 beats/minute.

His elevated serum hemoglobin A1C of 7.9% (normal 4.3 to 6.1%) was consistent with suboptimal diabetes control. He had a normal thyroid function screen and serum vitamin B12 levels. The results of tests for serum rapid plasma reagin, human immunodeficiency virus antibodies and a hepatitis panel were all negative. A screen for systemic vasculitides revealed an elevated anti-SSA antibody titer of 28.4EU/mL (reference range: neg <16EU/mL, equivocal 16 to 20EU/mL, positive >20EU/mL), with a normal anti-SSB titer of 0.3EU/mL (reference range: neg <16EU/mL, equivocal 16 to 20EU/mL, positive >20EU/mL). The results of the following serum or blood tests were negative, non-reactive or normal: anti-nuclear antibodies, anti-neutrophil cytoplasmic antibodies, complement 3 and 4 levels and rheumatoid factor titer.

Due to his demonstrable cranial nerve deficits associated with his severe brachial diplegia and orthostatic hypotension, an infectious, infiltrative or inflammatory disorder affecting his cranial nerves and cervical nerve roots was considered. Cerebrospinal fluid analysis revealed a normal white cell count of 1/μL (normal 0 to 5), with an elevated protein level of 81mg/dL (normal 15 to 45mg/dL), and glucose of 90mg/dL (normal 50 to 80mg/dL) with serum glucose of 160mg/dL. A mildly elevated immunoglobulin (Ig) G synthesis rate of 3.8mg/day was detected (normal -9.9 to +3.3 mg/day), suggesting increased intrathecal antibody production. Magnetic resonance imaging (MRI) of his brain with and without gadolinium contrast was normal. MRI of his spine revealed mild spinal canal stenosis at C5–C6 due to a small central disc protrusion without cord compression. No changes suggestive of spinal cord or nerve root inflammation were observed.

Nerve conduction studies (NCS; Table 2) suggested a primary axonal neuropathy. Mild conduction velocity slowing or prolonged distal latency, with reduced compound motor action potential amplitudes are consistent with this inference. There was no evidence of conduction block in any of the nerves studied. The normal tibial motor and sural sensory NCS provided evidence supporting the non-length-dependent nature of the patient’s axonal neuropathy. Monopolar needle electromyography revealed moderately severe chronic reinnervation changes in his radial, distal median, distal ulnar and axillary-innervated muscles bilaterally, with ongoing denervation changes in muscles innervated by his left distal median, right radial and bilateral axillary nerves only. Monopolar needle electromyography of cranial nerve VII innervated muscles revealed moderate chronic reinnervation changes bilaterally.

**Table 2:** Nerve conduction study data

**Sensory nerve conduction studies**			
**Nerve – Site**	**Amplitude (μV)**	**Distal latency (ms)**	**Conduction velocity (m/s)**
L Median – wrist	1.6 (≥10)	3.1 (≤2.8)	44.7 (≥50)
R Median – wrist	NR		
L Ulnar – wrist	7.5 (≥5)	2.9 (≤2.8)	48.0 (≥50)
R Ulnar – wrist	2.3 (≥5)	2.8 (≤2.8)	50.3 (≥50)
L Radial – forearm	7.3 (≥15)	2.0 (≤2.0)	50.0 (≥50)
R Radial – forearm	5.5 (≥15)	1.4 (≤2.0)	58.5 (≥50)
L Sural – calf	5.8 (>3)	3.2 (≤3.5)	43.3 (≥40)
**Motor nerve conduction studies**			
**Nerve-recording site – Stimulating site**	**Amplitude (mV)**	**Distal latency (ms)**	**Conduction velocity (m/s)**
L Median – APB/wrist	1.3 (≥5)	4.0 (≤4.5)	39.4 (≥50)
R Median – APB/wrist	2.3 (≥5)	4.9 (≤4.5)	42.6 (≥50)
L Ulnar – ADM/wrist	3.8 (≥5)	3.5 (≤3.5)	46.8 (≥50)
R Ulnar – ADM/wrist	4.0 (≥5)	3.4 (≤3.5)	51.4 (≥50)
L Radial – EIP/forearm	2.5 (≥2)	2.7 (≤2.9)	40.6 (≥50)
R Radial – EIP/forearm	3.4 (≥2)	2.6 (≤2.9)	47.6 (≥50)
L Facial – Nasalis/tragus	0.93 (≥1)	4.3 (≤4.2)	N/A
R Facial – Nasalis/tragus	1.8 (≥1)	3.5 (≤4.2)	N/A
L Tibial – AH/ankle	6.8 (≥3.5)	5.2 (≤6.0)	40.0 (≥40)

Normal values are indicated in parenthesis.

Key: ADM, abductor digiti minimi; AH, abductor halluces; APB, abductor pollicis brevis; EIP, extensor indicis proprius; L, left; N/A, not applicable; NR, not recordable; R, right.

The electrodiagnostic data was consistent with chronic, moderately severe, axonal mononeuropathies affecting the patient’s radial, median, ulnar, axillary and facial nerves; slightly worse on the left, as seen in mononeuropathy multiplex. There was also evidence of moderately severe chronic reinnervation changes affecting the L2–L4 myotomes on the left, suggestive of subclinical lumbar radiculopathies (explaining his diminished knee reflexes on examination). Secondary chronic inflammatory demyelinating polyradiculoneuropathy (CIDP) is unlikely because the clinical presentation, physical signs with preserved reflexes, were not consistent with CIDP, despite the elevated protein on cerebrospinal fluid (CSF). Moreover, the electrodiagnostic data did not meet the European Federation of Neurological Societies/Peripheral Nerve Society criteria for CIDP.

**Figure 1:**
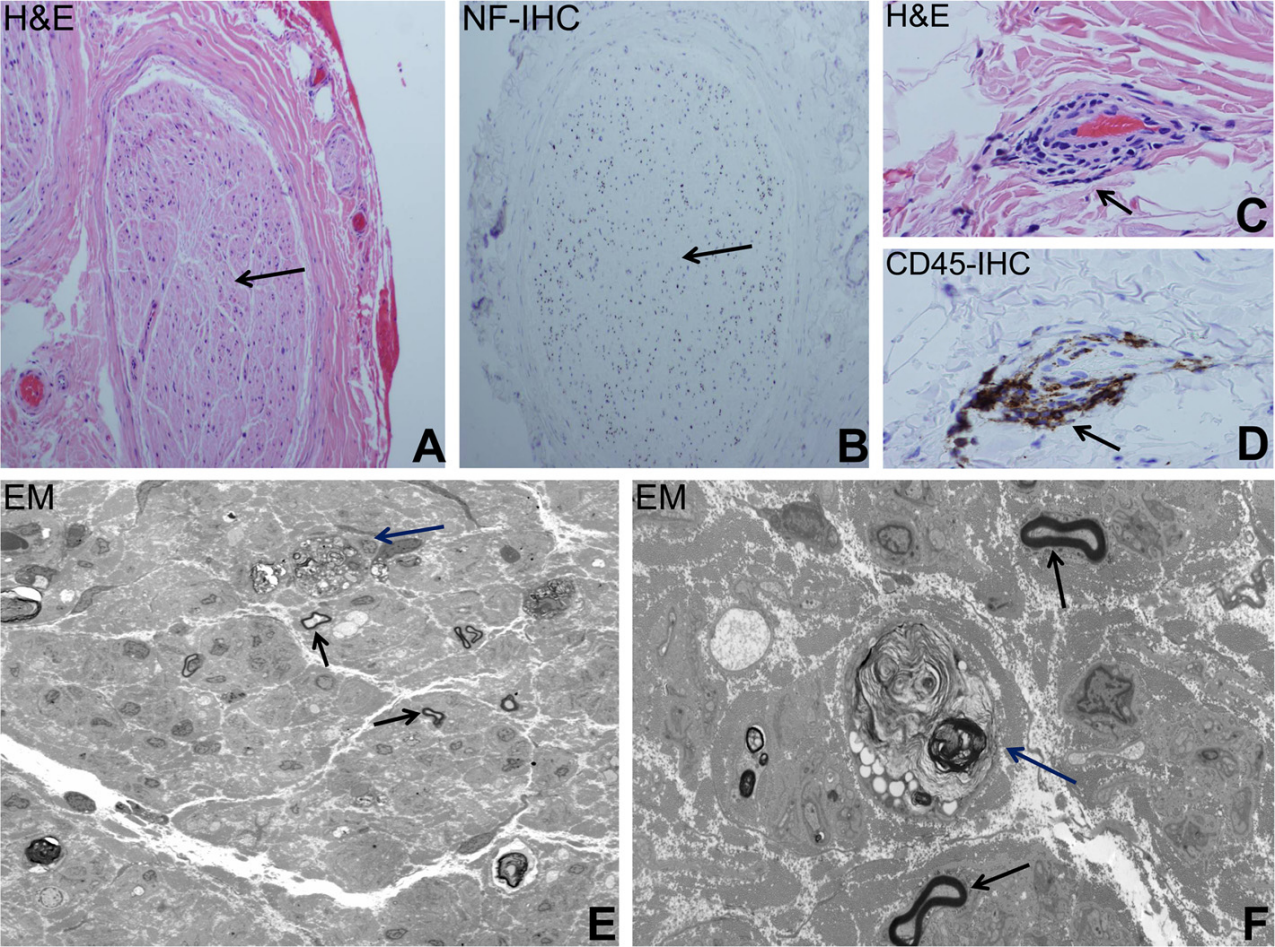
**Light and electron microscopy demonstrating vasculitic neuropathy.** Representative photomicrographs of fixed sections obtained from the left superficial radial nerve biopsy are shown. Hematoxylin and eosin (H&E)-stained sections demonstrate severe axonal loss with almost complete depopulation of myelinated axons in a nerve fascicle (black arrow), **(A)** supported by the lack of neurofilament (NF) staining (black arrow) on immunohistochemistry (IHC), as shown in **(B)**. H&E-stained sections show focal vasculitis involving small vessels with transmural lymphocytic infiltration in the epineurium (black arrow), **(C)**, verified by CD45 (also known as leukocyte common antigen)-positive mononuclear cell infiltrates on IHC (black arrow), **(D)**. Electron micrographs further demonstrate the severe axonal loss (~90 to 95% of large and small myelinated axons are no longer present) with residual myelinated axons (black arrows) and ongoing Wallerian degeneration (dark blue arrows) seen at lower **(E)** and higher magnifications **(F)**. Original magnifications: **A** and **B**: 20×, **C** and **D**: 64×, **E**: 1000× and **F**: 5000×.

He underwent a left superficial radial nerve biopsy that demonstrated a chronic vasculitic neuropathy with ongoing Wallerian degeneration, and severe end-stage axonal loss, as shown in Figure 1. This was consistent with a diagnosis of vasculitic mononeuropathy multiplex. A salivary gland biopsy (to evaluate for xerostomia) was normal. High-dose intravenous methylprednisolone was administered for 5 days followed by oral prednisone (1mg/kg/day) with strict glycemic control. A liberal salt diet, >2L fluid intake per day, JOBST^®^ waist high compression stockings (to provide 30 to 40mmHg pressure), and fludrocortisone were used to treat the severe orthostatic hypotension. The patient was discharged to a rehabilitation facility for upper extremity physical and occupational therapy.

At 3-month follow up, the patient reported mild improvement in his upper extremity function, evidenced by interval objective improvement on confrontational strength testing (Table 1) and improved facial expression. There was less orthostasis, with infrequent spells of postural lightheadedness despite non-compliance with compression stockings. The repeat MoCA score was unchanged, with stable deficits in visuospatial, executive function and delayed recall, although the patient and his family had noted a subjective improvement in his cognition. He was subsequently started on azathioprine, which was titrated upwards to a total daily dose of 2mg/kg/day alongside a gradual prednisone wean.

At 1-year follow up, he demonstrated further improvement in muscle strength (Table 1) with stable cognitive deficits. He had further improvement in orthostatic hypotension, and was continued on fludrocortisone. His glycemic control had worsened with a hemoglobin A1C of 10%, and continued with a dose tapering prednisone regimen in addition to azathioprine. His hemoglobin A1C 1.5 years after hospital discharge was 9.2% on low-dose prednisone and therapeutic azathioprine. He continues to receive specialist endocrinological care for diabetes. His neurological examination at his most recent evaluation was stable, with no appreciable change on confrontational muscle strength testing.

## Discussion

Our patient presented with progressive mononeuropathy multiplex, dysautonomia and cognitive decline, with a known history of diabetes mellitus that was suboptimally controlled. The electrodiagnostic studies and nerve biopsy were consistent with chronic axonal mononeuritis multiplex with ongoing nerve degeneration. Although diabetic radiculoplexus neuropathy is a rare cause of brachial diplegia [10], this clinical sign was attributed to mononeuropathy multiplex rather than bilateral brachial plexopathies, cervical radiculopathies or anterior horn cell disease based on the electrodiagnostic data. Distinguishing between brachial plexopathies and multiple mononeuropathies by electrodiagnostic testing can be challenging. The distinction depends on recognizing significant variability within individual nerves and nerves derived from the same trunk.

Our patient had severe orthostatic hypotension, with cardiovascular sympathetic α- and β-adrenergic dysfunction. Autonomic neuropathy is a well-recognized complication of diabetes, and may be found in isolation (preclinical or clinical) or in combination with diabetic polyneuropathy or other non-neurologic complications of diabetes. Up to 20% of diabetic patients may have cardiovascular autonomic dysfunction [2]. However, an autonomic neuropathy may also occur in patients with connective tissue disorders and vasculitis. Studies have shown a variable range (24 to 100%) of sympathetic and parasympathetic dysfunction in patients with autoimmune diseases [8]. In some cases, the autonomic neuropathy may be subclinical, or present as an initial clinical manifestation [8].

Finally, our patient also had moderate cognitive impairment on initial clinical presentation, involving frontal, parietal and temporal domains based on his MoCA data. A population-based study revealed that early onset, longer duration and greater severity of diabetes are associated with mild cognitive impairment. This cognitive decline was attributed to cerebral microvascular disease and subclinical infarctions [2]. The rate of cognitive decline observed in our patient raised the possibility for an alternative etiology. Although elevated CSF protein may be seen in diabetics, the elevated intrathecal IgG synthesis further raised our suspicion for an inflammatory or immune-mediated etiology. Cognitive impairment has been described in patients with small vessel vasculitis.

A small study reported that up to 30% of patients with small vessel vasculitis developed subclinical mild cognitive impairment, with mild abstract reasoning loss and non-verbal memory impairment being common manifestations [9,11]. Cognitive changes may be associated with an inflammatory encephalopathy. In support of this, frontal executive disorder, attention deficit and affective disorders have been described in Sjögren’s syndrome [9]. Recognizing clinical features that may support vasculitis rather than diabetic vasculopathy is important, as adjunctive immunosuppressant therapy would be required in the former and may worsen glycemic control without clinical benefit in the latter.

An extensive evaluation for systemic causes of vasculitis was unrevealing. Although peripheral nerve biopsies are invasive, this procedure is indicated when there is a high clinical suspicion for a non-systemic vasculitic neuropathy, in atypical diabetic neuropathies or suspected additional causes of neuropathy in diabetics [12]. Our patient met these criteria and the superficial radial biopsy confirmed the diagnosis of small vessel vasculitis; a diagnosis that significantly altered clinical management. Demonstrable clinical improvement was observed with chronic immunosuppressant therapy (over at least a year), in conjunction with adjunctive glycemic control and supportive pharmacotherapies and rehabilitation.

## Conclusions

We report an uncommon case of brachial diplegia, multiple cranial neuropathies, orthostatic hypotension and moderate cognitive impairment with electrophysiological evidence for chronic axonal mononeuropathy multiplex secondary to small vessel vasculitis in a diabetic patient. There was significant clinical improvement with high-dose immunosuppressant therapy. Mononeuropathy multiplex and vasculitis should be considered in diabetic patients irrespective of glycemic control status if these patients present with non-length dependent, asymmetrical motor or sensory deficits and progressive cognitive decline. Diagnostic confirmation is essential for the institution of appropriate therapies to prevent irreversible axonal loss and associated disability.

## Consent

Written informed consent was obtained from the patient for publication of this case report and accompanying images. A copy of the written consent is available for review by the Editor-in-Chief of this journal.

## Abbreviations

CIDP: Chronic inflammatory demyelinating polyradiculoneuropathy; CSF: Cerebrospinal fluid; Ig: Immunoglobulin; MAP: Mean arterial pressure; MoCA: Montreal Cognitive Assessment; MRI: Magnetic resonance imaging; NCS: Nerve conduction studies; PNS: peripheral nervous system.

## Competing interests

The authors declare that they have no competing interests.

## Authors’ contributions

SFZ and EEU obtained patient history and examination, and analyzed and interpreted the patient data regarding the brachial diplegia and vasculitis and were major contributors to writing the manuscript. JCG performed the histological examination and interpretation of the peripheral nerve biopsy. All authors read and approved the final manuscript.
